# Phylogenomic analysis reveals genome-wide purifying selection on TBE transposons in the ciliate *Oxytricha*

**DOI:** 10.1186/s13100-016-0057-9

**Published:** 2016-01-25

**Authors:** Xiao Chen, Laura F. Landweber

**Affiliations:** Department of Molecular Biology, Princeton University, Princeton, NJ 08544 USA; Department of Ecology and Evolutionary Biology, Princeton University, Princeton, NJ 08544 USA

**Keywords:** Transposable element, Transposon domestication, Ciliates, Genome rearrangement, Purifying selection

## Abstract

**Background:**

Transposable elements are a major player contributing to genetic variation and shaping genome evolution. Multiple independent transposon domestication events have occurred in ciliates, recruiting transposases to key roles in cellular processes. In the ciliate *Oxytricha trifallax*, the telomere-bearing elements (TBE), a Tc1/*mariner* transposon, occupy a significant portion of the germline genome and are involved in programmed genome rearrangements that produce a transcriptionally active somatic nucleus from a copy of the germline nucleus during development.

**Results:**

Here we provide a thorough characterization of the distribution and sequences of TBE transposons in the *Oxytricha* germline genome. We annotate more than 10,000 complete and 24,000 partial TBE sequences. TBEs cluster into four major families and display a preference for either insertion into DNA segments that are retained in the somatic genome or their maintenance at such sites. The three TBE-encoded genes in all four families display dN/dS ratios much lower than 1, suggesting genome-wide purifying selection. We also identify TBE homologs in other ciliate species for phylogenomic analysis.

**Conclusions:**

This paper provides genome-wide characterization of a major class of ciliate transposons. Phylogenomic analysis reveals selective constraints on transposon-encoded genes, shedding light on the evolution and domesticated functions of these transposons.

**Electronic supplementary material:**

The online version of this article (doi:10.1186/s13100-016-0057-9) contains supplementary material, which is available to authorized users.

## Background

Transposable elements (TEs) are genomic parasites present in all eukaryotic genomes. There exist multiple different classes of TEs, which occupy distinct fractions of the genome and show a wide variety of genomic activity. Despite the drastic differences, TEs play important roles in shaping the genome and facilitating genome evolution by processes that can promote genome rearrangements, contribute to the origin of new genes and alter gene expression [1–4].

Ciliates are unicellular eukaryotes that possess two types of nuclei, a transcriptionally active somatic nucleus and an archival germline nucleus [5]. The somatic nucleus develops from a copy of the germline through extensive genome rearrangements. In *Oxytricha*, the somatic macronucleus (MAC) is extremely gene dense, with ~16,000 short “nanochromosomes” that average 3.2 kb, and most encode a single gene [6]. The germline micronucleus (MIC), on the other hand, exhibits a highly fragmented and complex genome architecture, with short gene segments (Macronuclear Destined Sequences, MDSs) interrupted by brief noncoding sequences (Internal Eliminated Sequences, IESs). These DNA segments are the information that is retained in the soma after development; intriguingly, the DNA segments are often present in a permuted order or inverse orientation in the germline. Therefore, correct assembly of functional genes in the soma requires precise deletion of noncoding sequences and extensive reordering and inversion of gene segments that are “scrambled” in the germline. The somatic genome is free of transposons, although it contains some transposase-like genes [6]. Nearly 20 % of the germline genome is occupied by TEs [7], which are all eliminated during somatic development.

Ciliates provide novel model systems to study transposable elements because multiple TEs, especially the transposases they encode, have been recruited to provide important cellular functions for somatic development [8, 9]. The macronuclear genomes of *Tetrahymena* and *Paramecium* encode a homolog of the PiggyBac transposase that is expressed during development. Knockdown of the PiggyBac transposase results in a developmental defect, implicating its role in nuclear development [10, 11]. Tc1/*mariner* transposons are the most prevalent transposons in ciliate germline genomes, including the Tec elements in *Euplotes* [12] and *Tennessee, Sardine* and *Thon* elements in *Paramecium* [13, 14]. The terminal sequences of *Paramecium* IESs resemble the terminal inverted repeats of Tec elements in *Euplotes* [12, 15] and the ends of Tc1/*mariner* transposons [16], leading to the hypothesis that many IESs are remnants of TE insertions [17].

**Fig. 1:**
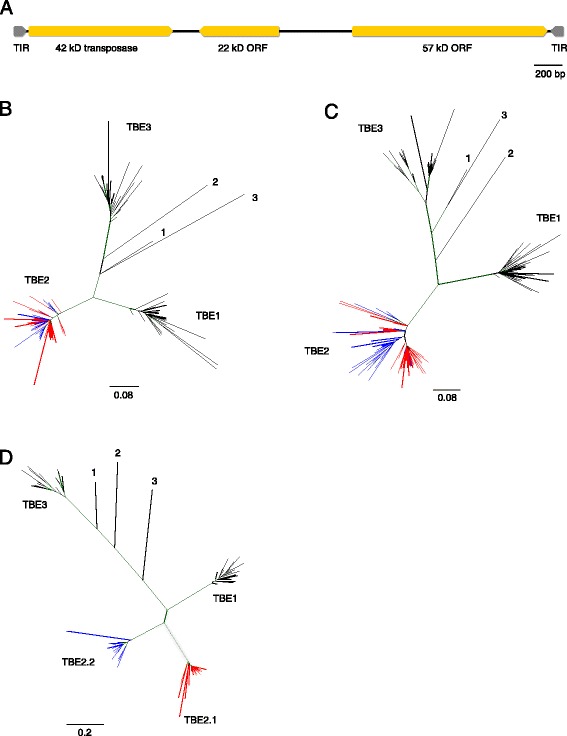
Phylogeny of sampled *Oxytricha* TBE genes and orthologs identified in three other stichotrich ciliates. **a** Schematic map of TBE transposons. Gray arrows represent terminal inverted repeats (TIR). Orange arrows represent ORFs encoded by TBEs. **b** Phylogeny constructed with TBE 42kD transposases (29 TBE1, 27 TBE2.1, 26 TBE2.2 and 25 TBE3 42kD protein sequences). Clades formed by TBE1, TBE2 and TBE3 are labeled accordingly. TBE2.1 representatives are indicated in red and TBE2.2 in blue. Internal branches supported by posterior probability higher than 0.9 are colored in green. **c** Phylogeny constructed with TBE 22kD ORFs (32 TBE1, 39 TBE2.1, 30 TBE2.2 and 28 TBE3 22kD protein sequences). Colors are as above. **d** Phylogeny constructed with TBE 57kD ORFs (27 TBE1, 26 TBE2.1, 23 TBE2.2 and 21 TBE3 57kD protein sequences). Clades formed by TBE1, TBE2.1, TBE2.2 and TBE3 are labeled accordingly; colors as above. The multiple sequence alignment was produced with MAFFT v6.956b and trimmed with trimAl v1.2 to remove excess gaps and poorly aligned regions. The unrooted Bayesian trees were produced with MrBayes v3.2.2 [35]. The three TBE orthologs are 1: *Sterkiella histriomuscorum*; 2: *Tetmemena sp.*; 3: *Laurentiella sp.*. All posterior probability values are above 0.5. The scale below the phylogeny illustrates branch substitutions per site

In *Oxytricha*, the telomere-bearing elements (TBEs) are another group of Tc1/*mariner* DNA transposons that have long been studied in ciliate germline genomes [18]. There is also phylogenetic evidence for recent insertion of TBEs [19]. TBEs encode three open reading frames (ORFs), a 42kD transposase, a 22kD ORF with unknown function and a 57kD ORF with zinc finger and kinase domains but unknown function (Fig. 1a). The 42kD transposase, together with the transposase encoded by *Euplotes* Tec elements and other Tc1/*mariner* transposases, belong to a superfamily of transposase genes with a common DDE catalytic motif [20]. Similar to the PiggyBac transposase, knockdown of the TBE transposase also leads to developmental defects, such as accumulation of unprocessed DNA and incorrectly rearranged nanochromosomes [21], suggesting that the TBE transposase has acquired an essential function in genome rearrangement. Because the transposase gene is present in many thousands of copies in the germline, this experiment was unique in knocking down such a high copy target. Nowacki *et al.* concluded that the 42kD transposase has likely been recruited for its DNA cleaving activity or another role in eliminating noncoding sequences, including their own elimination [21, 22].

A few studies have suggested that purifying selection is acting on the 42kD transposase encoded by TBEs [21, 23, 24]. However, these studies were limited by the small number (up to 100) of TBE sequences that were previously available. The levels of selection acting on the 22kD and 57kD ORFs have not been reported before and here we investigate their properties genome-wide. With the recent sequencing and assembly of the *Oxytricha* micronuclear genome [7], we are able to provide a thorough characterization of TBE sequences in the germline, including their genomic distribution and sequence features. We also infer the levels of selective constraints acting on the three transposon-encoded ORFs, and we discovered homologs of TBE transposons in other ciliate genomes. Together, these results provide insights into the origin and evolution of TBE transposons in *Oxytricha*.

## Results

### TBE sequences in the micronuclear genome cluster into four major families

We annotated TBE sequences in the micronuclear genome using the translated protein sequences of the three ORFs as query. In total we annotated 10,109 complete TBEs and 24,702 partial TBEs (Table 1, Additional file 1). The complete TBE sequences (those that encode all three ORFs) cluster into four major families, which correspond to the previously published TBE1 and TBE3 families [21], as well as two subfamilies within the TBE2 family. The two TBE2 subfamilies encode 42kD transposases and 22kD ORFs that are indistinguishable from each other, with comparable pairwise similarity either within or between TBE2.1 and TBE2.2 (Table 2); however, they encode distinct 57kD ORFs (% pairwise similarity 53.5 %, Table 2). Phylogenetic analysis confirms that the TBE2.1 and TBE2.2 42kD and 22kD genes do not form separate monophyletic clades (though there is some resolution of TBE2.1 and TBE2.2, especially for the 22kD gene, which may imply recent diversification) (Fig. 1b and c), whereas the 57kD genes are clearly distinguishable between TBE2.1 and TBE2.2 (Fig. 1d). The orientation of the three ORFs is consistent among the four TBE families, with the 22kD ORF in the reverse orientation relative to the other two ORFs (Fig. 1a). All TBEs contain a ~200 bp region with short tandem repeats between the 22kD ORF and the 57kD ORF.

**Table 1:** Genomic distribution of TBEs in the *Oxytricha* germline genome

	Complete								Partial			Total	
	#	Length (Mb)	% of complete TBEs by length	TIR			Near MDSs		#	Length (Mb)	% of partial TBEs by length	Length (Mb)	% of all TBEs by length
				2	1	0	#	% total copies					
TBE1	2502	9.9	24.75 %	2005	228	269	521	28.2 %	6216	6.5	24.8 %	16.4	24.8 %
TBE2.1	2484	10.0	25 %	2166	58	260	354	19.2 %	3129	3.9			
TBE2.2	1087	4.3	10.75 %	916	30	141	197	10.7 %	1146	1.3			
TBE2	3571	14.3	35.75 %	3082	88	401	551	29.9 %	9898 (TBE2.1 + TBE2.2 + unclassified partial TBE2)	10.5	40.1 %	24.8	37.5 %
TBE3	3946	15.8	39.5 %	3148	358	440	773	41.9 %	8588	9.2	35.1 %	25.0	37.7 %
Total	10,019	40.0	100 %	8235	674	1110	1845	100 %	24,702	26.2	100 %	66.2	100 %

**Table 2:** Pairwise percent protein sequence similarity of TBE genes

	42kD				22kD				57kD			
	TBE1	TBE2.1	TBE2.2	TBE3	TBE1	TBE2.1	TBE2.2	TBE3	TBE1	TBE2.1	TBE2.2	TBE3
TBE1	89.3 ± 4.4	74.2 ± 3.6	74.9 ± 3.3	67.1 ± 3.1	90.4 ± 4.2	67.1 ± 3.6	68.5 ± 3.6	65.3 ± 3.4	86.4 ± 5.2	46.3 ± 2.7	49.5 ± 2.8	36.4 ± 1.7
TBE2.1		89.3 ± 4.3	89.3 ± 4.2	67.9 ± 3.2		89.5 ± 5.1	87.6 ± 5.2	64.4 ± 3.4		87.2 ± 6.0	53.5 ± 3.1	34.5 ± 1.6
TBE2.2			90.8 ± 4.5	68.7 ± 2.9			89.8 ± 4.8	65.5 ± 3.1			86.1 ± 5.9	38.2 ± 1.8
TBE3				90.9 ± 4.8				89.9 ± 5.4				86.1 ± 7.5

Most annotated, complete TBEs are flanked by two terminal inverted repeats (TIRs) (Table 1). Apart from differences in the sequences of the three ORFs, the four TBE families also have distinct TIRs, with variation in both sequence and length (Table 3). All TIRs contain the *Oxytricha* telomeric repeat, CA_4_C_4_A_4_C_4_, with the exception of TBE2.1 which contains CA_4_C_4_A_4_C_3_. TBE2.2 transposons have two distinct types of TIRs, one of which is a 21 bp shorter version of the TBE2.1 TIR. The two TBE2.2 TIRs (117 bp and 112 bp) are 92.5 % similar to each other. The protein sequences of TBE2.2 transposons with these two TIRs are indistinguishable from each other (percent pairwise sequence similarity between 57kD genes of the two types: 87 ± 4.3, vs. 86.6 ± 5.9 and 89.2 ± 6.3 % within each group). Each family also exhibits unique distances between the TIR and the start of the first ORF (42kD) and between the TIR and the end of the last ORF (57kD) (Table 3). Curiously, the TIR of TBE2.1 ends right before the start codon of the 42kD ORF. For the two types of TIRs within TBE2.2, although they are shorter than the TIR of TBE2.1, the distance between the end of TIR and the 42kD ORF is longer, such that the total distance between the 5′ terminus of a TBE2 and the start of the 42kD ORF is precisely the same among most TBE2 sequences. It is possible that in the TBE2.2 subfamily, the selective constraints on TIRs are weaker so that the TIR becomes shorter, leaving the sequence between the TIR and the start of the 42kD ORF more flexible to accumulate substitutions.

**Table 3:** Features of *Oxytricha* TBEs and complete TBEs identified in other stichotrich genomes

		Terminal inverted repeat (TIR) (Underline for telomeric repeat)	Target site duplication	Distance (bp) between (mode, % of mode)	
				TIR/42kD	57kD/TIR
*Oxytricha trifallax*	TBE1	CAAAACCCCAAAACCCCTTAATGAGGTTTA	ANT	3	41
		TAAGTGCTTTGATTTGTAGGGAATTTGTTA		97.7 %	86.5 %
		GGGGTTGGGGTTATTAAT (78 bp)			
	TBE2.1	CAAAACCCCAAAACCCTTTCAGTAGTTTGA	ANT	0	23
		TTGAGTTTTTGATTGATAAAAGTAGACTAT		99.1 %	82.3 %
		TAGTGCATACTTTATTAGGGTTTTAATAGG			
		GTTTATGTAGGGGTTTAATGTTTAAATATT			
		AGTAATTTAAGTGAGTAT (138 bp)			
	TBE2.2	CAAAACCCCAAAACCCTTTCAGTAGTTTGA	ANT	21	44
		TTGAGTTTTTGATTGATAAAAGTAGACTAT		96.4 %	86 %
		TAGTGCATACTTTATTAGGGTTTTAATAGG			
		GTTTATGTAGGGGTTTAATGTTTAAAT (117 bp)			
		CAAAACCCCAAAACCCCTGAAGTTGTTTGA	ANT	26	49
		TTGAGTTTTTGATTGATGAAAGTAGACTAT		97.6 %	92.1 %
		TAACGCATGCTTTATTAGGGTTTTAATAGG			
		GTTTATGTAGGGGTTTAGGGTT (112 bp)			
	TBE3	CAAAACCCCAAAACCCCTTAGTGAGGTTTA	ANT	17	54
		TAAGTGCTTTGATTTGTAGGGTATAGTTGG		93.3 %	84.6 %
		GGTCTTATTGGGGTTAGTAGAGAAA (85 bp)			
*Sterkiella histriomuscorum*		CAAAACCCCAAAACCCCTTCATGAGTTGTT	ANT	0	28
		TATGAGTTTTTGATTGTGTTGGGATTATTA			
		GTGTTTATTAGGGTTTATTAATAATTGGGG			
		TTAGTACACAAA (102 bp)			
*Tetmemena sp.*		CAAAACCCCAAAACCCCATAATATGATAAG	ANT	−4	−6
		AAAGTGAAAATAAGTTGTGTATAATTAATT			
		TCTTTATTAATACTTATAATCATGC (85 bp)			
*Laurentiella sp.*		CCCAAAACCCCAACTACTTATAAAATGTGA	ANT	1	11
		TTAATAATAAGAATTGATATATATTAATTT			
		CATAATTATCAACGTTTTTAGAGTAATTAA			
		ACTGCGATGAGTTATATAAA (110 bp)			

### Distribution of TBEs in the micronuclear genome

Annotated TBEs occupy ~13.3 % of the micronuclear genome. This is slightly smaller than the previously reported estimate of 15 % [7] because the previous annotation is based on RepeatMasker (http://www.repeatmasker.org/), which uses sequence similarity at the nucleotide level, often including short nucleotide matches. Here, our annotation approach is based on sequence similarity at the protein level, and therefore sequences other than the three ORFs, such as terminal inverted repeats and spacer regions between the three ORFs, may have been missed, especially for partial TBEs. Among all annotated TBEs, 24.8 % are TBE1, 37.5 % are TBE2, with the ratio between TBE2.1 and TBE2.2 approximately 2.3:1, and 37.7 % are TBE3 (Table 1).

Annotated partial TBEs are more likely to be located within 500 bp of contig ends (57.7 %) than complete TBEs (19.4 %), suggesting that the original PacBio and Illumina-based genome assembly algorithm [7] had difficulty spanning repetitive sequences. Therefore, improvements in the genome assembly would be expected to lead to completion of these terminal, partial TBEs. On the other hand, partial TBEs located internal to a contig have lower sequence similarity to the protein sequence consensus of each family than those at contig termini (for example, % protein sequence similarity for internal partial TBE1 42kD genes: 71.6 ± 18.5; vs. terminal partial TBE1 42kD genes: 90.1 ± 9.3), suggesting that a significant portion of internal, partial TBEs are degenerate copies that are truly partial TBEs due to loss of one or two ORFs.

TBE sequences display a preference for insertion into MDSs (precursor DNA segments that are incorporated into the somatic genome), with more frequent distribution near MDSs (18.3 % within 500 bp) than the 11.1 % estimate of the genome space occupied by MDSs (Table 1, Chi-squared test, *p*-value = 6.304e-05). The short noncoding elements (IESs) that interrupt MDSs have long been proposed to be remnants of ancient transposon insertions [17]. Since the TBE transposase has been implicated in IES removal and genome rearrangement [21], this enrichment near MDSs may facilitate the removal of both the transposons, themselves, and IESs. Among the TBEs that are near MDSs, there is a slight enrichment for members of TBE1 and TBE3, accompanied by a slight depletion of both TBE2 representatives (Table 1). Satellite repeats are another major class of repetitive sequences in the germline genome. There is no significant preference for TBE insertions near satellite repeats. Only 80 (0.79 %) complete TBEs reside within 500 bp of 380 bp repeats (which occupy 1.4 % of the genome) and 97 (0.96 %) complete TBEs reside near 170 bp repeats (which represent 1.2 % of the genome). Therefore, TBEs are more often associated with MDSs. Either their preferential insertion or maintenance near MDS-rich regions is consistent with the inferred participation of TBEs in genome rearrangement events that reassemble MDSs [21].

### Sequence analysis of TBE sequences

Complete TBE sequences are highly similar to each other within each family (Table 2), with ~90 % pairwise similarity for the 42kD and 22kD ORFs and a slightly lower similarity, ~86 %, for the 57kD ORF. This high sequence similarity suggests that either their expansion and insertion occurred relatively recently or that each family is subject to strong selective constraints. TBE1 and TBE2 members are more similar to each other than to TBE3 (Table 2). Among different families, the 42kD transposase gene is more conserved than the 22kD ORF. The 57kD ORF is the least conserved compared to the other two ORFs (Table 2), with just 36.4 % similarity between TBE1 and TBE3, for example.

The terminal inverted repeat sequences are highly similar between both ends of a TBE (% sequence similarity: TBE1: 95.4 ± 3.6; TBE2.1: 93 ± 4.4; TBE2.2: 93.2 ± 4.4; TBE3: 95.9 ± 3.7), also consistent with either recent insertion or selective constraint.

We observe a prevalence of premature stop codons and frameshifts in TBE open reading frames (Table 4). 1360 TBE1, 1025 TBE2.1, 503 TBE2.2 and 1842 TBE3 elements encode three full-length proteins, but 96–98 % of these transposons contain premature stop codons and/or frameshifts in at least one of the three genes. The prevalence of stop codons is particularly prominent in the TBE3 42kD and 22kD ORFs, with an excess of stop codons occurring at a few specific sites. Among all TBE3 42kD genes (352 residues), 83.5 % contain a stop codon at residue 70, and 13.8 % contain a stop codon at residue 127. Among all TBE3 22kD genes (192 residues), 35.2 % contain a stop codon at residue 38, 22.6 % contain a stop codon at residue 39, and 83.2 % contain a stop codon at residue 186. While the prevalence of stop codons and frameshifts could be an artifact of less accurate genome assembly in repetitive regions, the enrichment of stop codons in TBE3s cannot be explained by such an assembly artifact alone, since assembly errors would result in stop codons that are randomly distributed across the coding sequence rather than enriched at specific sites.

**Table 4:** Prevalence of premature stop codons and frameshifts

	42kD		22kD		57kD	
	Stop codon	Frameshift	Stop codon	Frameshift	Stop codon	Frameshift
TBE1	30.4 %	76.8 %	14.9 %	59.0 %	38.1 %	87.1 %
TBE2.1	29.6 %	79.1 %	23.1 %	62.7 %	45.5 %	86.2 %
TBE2.2	29.4 %	74.8 %	16.8 %	60.8 %	36.7 %	87.3 %
TBE3	84.7 %	75.7 %	90.7 %	56.3 %	35.8 %	81.3 %

**Fig. 2:**
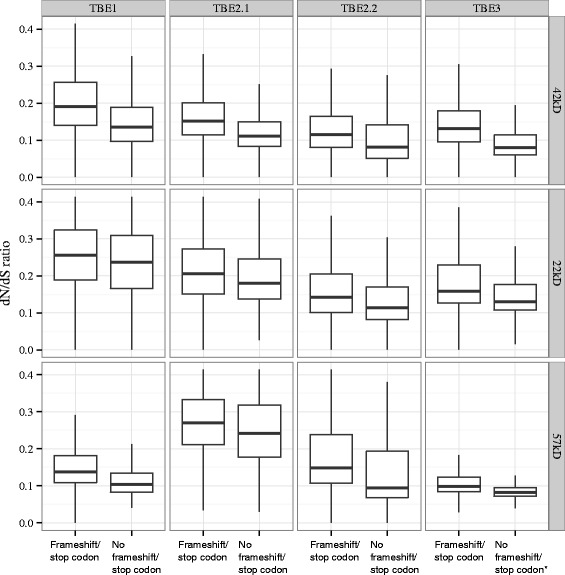
TBE substitution rate variation. Box plots represent dN/dS values for the three TBE-encoded genes (with or without premature stop codons or frameshifts) among the four TBE families. The numbers of ORFs analyzed are summarized in Additional file 2. *For TBE3 42kD and 22kD genes, since very few sequences lack frameshifts or premature stop codons, we permitted the presence of the most frequent stop codons at residue 70 (42kD protein) and residue 186 (22kD protein) for the “No frameshift/stop codon” group

Substitution rate analysis suggests that both groups of TBE sequences that do or do not contain stop codons or frameshifts have dN/dS ratios significantly lower than 1 (Fig. 2, Additional file 2). The overall dN/dS ratios are in the range of 0.1–0.3, suggesting genome-wide purifying selection acting on TBEs, which is consistent with earlier small-scale studies on the 42kD transposase [21, 23, 24] and unpublished studies from our lab of the other two ORFS [25]. Our study demonstrates that purifying selection is also acting on the other two TBE-encoded ORFs, indicating potential functional roles of these two genes. TBEs without a premature stop codon or frameshift display lower dN/dS values than those that contain premature stop codons or frameshifts and thus are more likely to be functional transposon copies. For TBE3 42kD and 22kD genes, since very few copies lack stop codons (Table 4), we included in the “No frameshift/stop codon” group those sequences that contain just the most abundant stop codons listed above. This category also displays lower dN/dS ratios than those with other stop codons or frameshifts. In addition to pairwise dN/dS analysis, we also compared likelihoods of evolutionary models with estimated dN/dS ratios <1 and with dN/dS fixed at 1 (no selection) using a chi-squared test (Additional file 3). The former model fits significantly better in every case. The observed levels of purifying selection acting on TBE proteins that contain stop codons or frameshifts, especially the TBE3 42kD and 22kD genes, may suggest the presence of a biological mechanism to correct the stop codons and frameshifts so that functional proteins can be expressed.

### TBEs in newly sequenced stichotrich genomes

We searched six newly sequenced stichotrich macronuclear genomes [26] for orthologous sequences of TBE transposons. Since TBEs are repetitive sequences that occupy a large portion of the micronuclear genome, their copy number in whole cell DNA is often comparable to nanochromosomes at high copy number in the macronuclear genome. DNA prepared from whole cell extracts therefore often contains some TBE sequences. We took advantage of this to extract TBE orthologs from macronuclear genome assemblies prepared from whole cell DNA.

We were able to identify complete or partial TBE sequences in the macronuclear genome assemblies of *Urostyla sp.*, *Paraurostyla sp.*, *Laurentiella sp.*, *Stylonychia lemnae*, *Tetmemena sp.* and *Sterkiella histriomuscorum* (the phylogeny of these species is discussed in [26]). Complete TBEs were found in *Laurentiella*, *Tetmemena* and *Sterkiella* (Table 3), with conserved orientation of the three ORFs but distinct terminal inverted repeats. Complete but degenerate TBEs were found in *Paraurostyla*, with no terminal inverted repeat and an inverted 57kD ORF. In *Stylonychia*, we could only identify incompletely assembled contigs containing TBE sequences. In *Urostyla*, we found only one sequence that exhibits weak protein sequence similarity to the *Oxytricha* 42kD transposase and we identified no homolog for the 22kD and 57kD ORFs. Similar to the DDE transposases in *Euplotes* Tec elements and the *Tetrahymena* and *Paramecium* genomes [9], the *Urostyla* DDE transposase homolog is very divergent from the *Oxytricha* 42kD transposase, exhibiting ~26 % sequence similarity in only a ~100 amino acid region containing the DDE motif towards the C-terminus.

Phylogenetic analysis supports the grouping of the assembled TBE orthologs in *Sterkiella, Tetmemena* and *Laurentiella* with *Oxytricha* TBE3 (Fig. 1b, c and d) (the incompletely assembled TBE sequences in the *Urostyla*, *Paraurostyla* and *Stylonychia* genomes also group with TBE3, data not shown). We found no premature stop codon in *Sterkiella* TBE orthologs of the 42kD and 22kD ORFs, the *Tetmemena* ortholog of the 22kD TBE ORF and the *Laurentiella* ortholog of the 22kD ORF. The *Tetmemena* and *Laurentiella* orthologs of the 42kD transposase both contain a premature stop codon, neither of which is present at a homologous position with each other nor with common sites of premature stop codons in the 42kD ORFs of *Oxytricha* TBE3. This suggests that the most common premature stop codons in the *Oxytricha* TBE3 genes may be specific to the *Oxytricha* lineage.

Since TBE orthologs group with TBE3, and TBE1 and TBE2 are more similar to each other than either is to TBE3 (Table 2), we infer that the TBE1 and TBE2 divergence and expansion most likely occurred recently in the *Oxytricha* lineage. Alternatively, the divergence may have occurred earlier but orthologous TBE1 and TBE2 sequences could be rare, or otherwise absent from the whole cell genome data for all other stichotrich genomes surveyed, or TBE 1 and 2 could have been lost from those lineages during evolution; however, these are all less parsimonious explanations than the conclusion that TBE 1 and 2 arose after TBE3 and underwent an expansion in the *Oxytricha* lineage. Furthermore, preliminary micronuclear genome sequence data from one of the outgroup species confirm the absence of TBE1/TBE2 orthologs in its micronuclear genome (Beh, Lindblad, Chen, Sebra, and Landweber, unpublished). Since the micronuclear genome sequences of most stichotrichs are not available and the DDE transposases in *Euplotes*, *Tetrahymena* and *Paramecium* are too divergent to provide outgroups, it is difficult to infer features of the ancestral TBE transposon that first invaded stichotrich germline genomes.

## Discussion

We report a genome-wide characterization of the distribution and sequence features of TBE transposons in *Oxytricha* and provide phylogenomic evidence that the root among them may be in the TBE3 clade. The four major TBE families each have distinct terminal inverted repeats and spacer regions between TIRs and ORFs.

Of the three TBE-encoded genes, the 57kD ORF is much less conserved among different families than the 42kD and 22kD ORFs. It is possible that the structure and function of the 57kD protein allows it to be tolerant to more substitutions. Notably, the two subfamilies of TBE2 have similar 42kD and 22kD genes but very different 57kD genes, consistent with both increased variation in and the possible expanding roles of the 57kD protein. One type of the TBE2.2 terminal inverted repeat is precisely 21 bp shorter than the TBE2.1 TIR. It is possible that TBE2.1 is the ancestral form of TBE2 and that TBE2.2 diverged later from TBE2.1 with the acquisition of substitutions in the 57kD ORF, and that this was accompanied by shortening or altering the TIRs. While all three ORFs currently appear to be under purifying selection, the branch lengths in Fig. 1d suggest that the 57kD gene appears to have evolved rapidly under relaxed selective constraints after the divergence of the TBE families. This may have been a period when the diversification of the 57kD genes contributed to the functional differences among TBE families. Functional studies of the 57kD protein would provide insight into its biological roles in transposon elimination or genome rearrangement.

No TBE1 or TBE2 orthologs are found in related stichotrich ciliates, but future sequencing of their germline genomes would provide a better view of their germline transposons and help delineate the origin and evolution of TBE1 and TBE2, as well as TBE2.1 and TBE2.2 elements. Comparative germline genome sequences will also shed light on the evolutionary relationship between TBE3 and TBE1/2, and possibly permit inference of the ancestral TBE type that first invaded ciliate genomes.

Our analysis of transposons relies on the accuracy of genome assembly. The *Oxytricha* micronuclear genome was assembled using a hybrid approach, taking advantage of long PacBio reads that average ~7 kb [7]. A complete TBE sequence is ~4 kb and can be easily spanned by a PacBio read. Therefore, the accuracy of the assembly should be high for characterization of the genomic location and distribution of TBEs. However, PacBio reads were first error-corrected with high confidence unitigs assembled from Illumina reads before genome assembly [7], and Illumina reads, limited by their short length, can be ambiguous in repetitive regions. While Illumina unitigs are longer and more informative than Illumina reads, it is still possible that unitigs were ambiguous in resolving individual repeats, and hence that some PacBio reads deriving from repetitive regions may have not been corrected 100 % accurately. Therefore, TBE sequences will assemble less accurately than non-repetitive regions. The observed prevalence of premature stop codons and frameshifts may partially derive from this assembly artifact. However, such assembly artifacts could not explain the enrichment of stop codons at specific sites in the 42kD and 22kD ORFs of TBE3, since they would result in stop codons that are randomly distributed across the coding sequence. Assembly artifacts may have also contributed to the slightly higher dN/dS ratios that we identified, compared to ref. [21]. Another factor contributing to the higher dN/dS ratios could be that we included all annotated TBEs (both active and inactive copies) in the analysis, whereas the previous study was based on a small set of known TBE sequences that are more likely to contain active copies.

## Conclusions

This study provides the first genome-wide evolutionary analysis of ciliate transposons, suggesting the importance of all three TBE-encoded gene products, either in genome arrangement or other aspects of late nuclear differentiation, when the transposon genes are expressed. Sequencing and comparative analysis of more ciliate germline genomes will provide insights into the evolution and recruitment of domesticated transposons in genomes with complex genetic architecture.

## Methods

### Annotation and extraction of TBE sequences from the micronuclear genome

The protein sequences for the three ORFs (GenBank accession: AAB42034.1, AAB42016.1 and AAB42018.1) were used to query the *Oxytricha* micronuclear genome (GenBank accession: ARYC00000000) as well as the ciliate macronuclear genome assemblies (*Urostyla sp*.: LASQ02000000, *Paraurostyla sp.*: LASR02000000, *Laurentiella sp.*: LASS02000000, *Sterkiella histriomuscorum*: LAST02000000*, Tetmemena sp.*: LASU02000000 *and Stylonychia lemnae*: ADNZ03000000) with TBLASTN (BLAST+ [27], parameters: -db_gencode 6 -evalue 1e-7). TBE regions were annotated according to the TBLASTN output. Regions containing three ORFs in proximity (within 1 kb from each other) and in the correct orientation were annotated as complete TBEs, while those that do not contain all three ORFs were annotated as partial TBEs.

### Clustering and alignment of TBE sequences

Complete TBE sequences were aligned to each other using an all-by-all BLASTN (BLAST+ [27], parameters: -word_size 50). Pairwise sequence similarity values were converted into input for MCL (parameter: -I 1.2) [28], which clustered TBE sequences into large clusters. Coding sequences were extracted using Exonerate [29] (parameters: --model protein2dna --geneticcode 6 --ryo “ > %ti_%tab_%tae\n%tcs” --verbose 0 --showalignment no --showvulgar no). All-by-all BLASTP searches were performed on translated protein sequences (BLAST+ [27], E-value cutoff 10^−7^) and pairwise protein sequence similarities were extracted from the BLASTP output. Terminal inverted repeats were determined by aligning the two ends of a TBE sequence using BLASTN, and clustering and consensus sequence generation were performed using UCLUST [30]. For *Oxytricha* TBEs, target site duplications were determined by comparing the MIC genome sequences immediately flanking TBEs. For TBEs identified in other ciliate macronuclear genomes, target site duplications were determined by mapping genomic reads to TBEs using BWA [31] (default parameters) and comparing the sequences flanking the terminal inverted repeats.

### Construction of phylogenetic trees

Randomly sampled protein sequences of the three TBE ORFs were aligned with MAFFT [32] and excess gaps and poorly aligned regions were removed with trimAl (version 1.2, with the “-automated1” parameter) [33]. We used ProtTest [34] to determine the most suitable protein model (JTT + I + G). Phylogenetic trees were generated from the alignments using MrBayes v3.2.2 [35] (parameters: prset aamodelpr = fixed(jones); lset rates = invgamma). Trees were drawn using FigTree 1.4.2 (http://tree.bio.ed.ac.uk/software/figtree/).

### Estimation of substitution rates

Pairwise protein alignments (MAFFT version 6.956b, [32]) were performed for each of the three genes (stop codons masked and frameshifts corrected) encoded by TBEs. Protein alignments were converted to coding sequence alignments using PAL2NAL [36]. The lengths of trimmed alignments are 344 codons (42kD protein), 187 codons (22kD) and 471 codons (57kD). Nonsynonymous to synonymous rate (dN/dS) ratios were calculated using the codeml program in PAML [37] (version 4.5) with parameters “icode = 5, runmode = −2, CodonFreq = 2”. Synonymous substitution rates below 0.01 or above 5 were excluded from the analysis. In addition to pairwise dN/dS estimation, we randomly sampled 50 to 80 42kD, 22kD and 57kD ORFs and used codeml to compare likelihoods of models with estimated dN/dS (runmode = 0, fixed_omega = 0) and that with dN/dS = 1 (runmode = 0, fixed_omega = 1, omega = 1) (Additional file 3).

## Additional files

Additional file 1:
**Annotation of TBE sequences in** ***Oxytricha*** **micronuclear genome in gff format.** (GFF 2.38 mb) Additional file 2:
**Number of 42kD, 22kD and 57kD ORFs included in the pairwise dN/dS analysis.** (XLSX 38.5 kb) Additional file 3:
**Likelihood comparison of models with dN/dS=1 and dN/dS<1.** (XLSX 41.4 kb)

## Abbreviations

TE: Transposable elements
TBE: Telomere bearing elements
MDS: Macronuclear destined sequence
IES: Internal eliminated sequence
ORF: Open reading frame
TSD: Target site duplication
TIR: Terminal inverted repeat
